# Temporal metabolic profiling of bone healing in a caprine tibia segmental defect model

**DOI:** 10.3389/fvets.2022.1023650

**Published:** 2023-01-17

**Authors:** Austin J. Bow, Rebecca E. Rifkin, Caitlin Priester, Courtney J. Christopher, Remigiusz M. Grzeskowiak, Silke Hecht, Steve H. Adair, Pierre-Yves Mulon, Hector F. Castro, Shawn R. Campagna, David E. Anderson

**Affiliations:** ^1^Department of Large Animal Clinical Sciences, University of Tennessee College of Veterinary Medicine, Knoxville, TN, United States; ^2^Department of Animal Science, University of Tennessee, Knoxville, Knoxville, TN, United States; ^3^Department of Chemistry, University of Tennessee, Knoxville, Knoxville, TN, United States; ^4^Department of Small Animal Clinical Sciences, University of Tennessee College of Veterinary Medicine, Knoxville, TN, United States; ^5^Biological and Small Molecule Mass Spectrometry Core and the Department of Chemistry, University of Tennessee, Knoxville, Knoxville, TN, United States; ^6^University of Tennessee College of Veterinary Medicine, Knoxville, TN, United States

**Keywords:** bone tissue engineering, metabolomics, large animal model, biomarkers, bone remodeling, caprine, regenerative medicine, veterinary research

## Abstract

Bone tissue engineering is an emerging field of regenerative medicine, with a wide array of biomaterial technologies and therapeutics employed. However, it is difficult to objectively compare these various treatments during various stages of tissue response. Metabolomics is rapidly emerging as a powerful analytical tool to establish broad-spectrum metabolic signatures for a target biological system. Developing an effective biomarker panel for bone repair from small molecule data would provide an objective metric to readily assess the efficacy of novel therapeutics in relation to natural healing mechanisms. In this study we utilized a large segmental bone defect in goats to reflect trauma resulting in substantial volumetric bone loss. Characterization of the native repair capacity was then conducted over a period of 12 months through the combination of standard (radiography, computed tomography, histology, biomechanics) data and ultra-high-performance liquid chromatography-high resolution mass spectrometry (UHPLC-HRMS) metabolic profiling. Standard metrics demonstrated that samples formed soft callus structures that later mineralized. Small molecule profiles showed distinct temporal patterns associated with the bone tissue repair process. Specifically, increased lactate and amino acid levels at early time points indicated an environment conducive to osteoblast differentiation and extracellular matrix formation. Citrate and pyruvate abundances increased at later time points indicating increasing mineral content within the defect region. Taurine, shikimate, and pantothenate distribution profiles appeared to represent a shift toward a more homeostatic remodeling environment with the differentiation and activity of osteoclasts offsetting the earlier deposition phases of bone repair. The generation of a comprehensive metabolic reference portfolio offers a potent mechanism for examining novel biomaterials and can serve as guide for the development of new targeted therapeutics to improve the rate, magnitude, and quality of bone regeneration.

## 1. Introduction

As a complex and dynamic organ, bone observes a constant state of scalable remodeling, culminating from intricate cell-cell and cell-matrix signaling, to maintain structural stability. Traumatic injury of this tissue stimulates a cascade of repair events that rely on recruiting progenitor cells to the site and increasing the mineral deposition, resulting in a callus structure that can then be gradually resorbed and remodeled (1, 2). However, this process can be adversely complicated by injuries in which large bone volume has been lost, with the proximal and distal bone surfaces unable to effectively communicate over this distance. For such cases a graft technology is required to facilitate the bridging of fracture ends and prevent progression to fracture non-union (3). The current gold standards for bone grafting material have been cortical and cancellous autografts from a donor site of the individual or the allografts derived from cadaveric tissue (4, 5). However, this procedure is not without inherent risks and ~30–60% of grafting procedures result in one or more complications, which range from infection and incomplete integration to donor site morbidity (6). In humans, the incidence of non-union fractures is ~5–10%, though definitions for delayed healing or fracture non-unions are still inconsistent and subjective (7). The prevalence of fractures appears to be closely associated with increased age due to the increasing brittleness of aging bone, as well as lifetime comorbidities such as diabetes and obesity (8). In the United States, annual expenditures related to fracture repair is estimated at ~$200 billion, with over 500,000 cases requiring a bone grafting material. Additionally, musculoskeletal injuries in soldiers have been reported to compromise approximately 50% of all combat-related wounds, further exacerbating this financial burden and highlighting the need for new and innovative regenerative therapies (9). Specifically, the development of a novel osteobiologic technologies capable of demonstrating similar or improved efficacy compared with current standards of care and that can be readily manufactured represents a primary goal in the field of bone tissue engineering (10).

Few studies have examined bone repair and regeneration in animal models exceeding 90 days, with even fewer implementing large animal models for increased translation to human medicine largely stemming from increased costs associated with use of these models (11–14). To address criteria for new medical devices, such as novel osteobiologic technologies, as set by the Food and Drug Administration and International Organization for Standardization [ISO] 10993, a relevant preclinical segmental bone defect in a large animal model is required to ensure the safety and efficacy of novel devices (6). Goats and sheep represent ideal animals for the study of bone regeneration as the sequence of cellular events during osseointegration of grafting material has been shown to be similar to humans (15). Historically in the evaluation of a new biomaterial, tibial fracture gap sizes have been made with a length of 2.0–2.5 times that of the shaft diameter, resulting in ostectomies ranging from 20 to 30 mm in length (16). Stabilization of these defects for the duration of the study has been attained using a variety of fixation methods, including external fixator pins, bone plates, and intramedullary nails.

Although numerous biomaterials have been designed for bone tissue, a key challenge for comparing their osteogenic repair potential continues to be an insufficient characterization of the biological landscape during the natural repair process (17). Specifically, robust profiling of the myriad of small molecules, whose fluctuations regulate cell signaling and metabolic functions, appears to be limited in published literature (18). This is particularly evident when searching for information on organisms beyond commonly studied species such as human, rat, and mouse systems. Currently the metrics for assessing such studies rely on radiography, computed tomography, biomechanics, and histology. These technologies are capable of broadly comparing treatments but may be bolstered substantially by supplementation with bioinformatic data (19). This offers the potential to develop a panel of associated biomarkers to serve as sensitive predictive measures for a grafts outcome even at early stages following implant. Furthermore, this data can be implemented during design stages to generate advanced biomaterials capable of eliciting a tailored cell response (20).

The establishment of biomarkers relating to repair efficacy of large-scale bony defects would dramatically enhance the ability to both precisely and objectively rank novel emerging biomaterials, particularly those implementing biological additives such as stem cells (21). In order to elucidate promising biomarker candidates for these graft technologies, it is prudent to examine molecular profiles for biological samples taken from the defect region over a time dependent study frame. This baseline data can then be mined to determine correlative patterns relating to repair, allowing for the establishment of a reference template. To minimize anomalies within this reference portfolio, enhanced numbers of both technical and biological replicate samples are crucial and can help to bolster the predictive capacity of a given marker panel (22).

Metabolomics is a quickly emerging field of science that combines statistical and chemistry-based methods of analysis. Metabolites, endogenous molecules smaller than 1,500 Da such as amino acids, peptides, nucleic acids, lipids, and alcohols, comprise the metabolome and display unique profile characteristics that reflect intricate pathway signaling processes within organisms (23, 24). The metabolic profile provides direct insight into the physiological state as these small molecules are an essential aspect for basic physiological functions. Their fluctuation patterns can be investigated in biological samples including solid tissue biopsies or fluid samples to establish effective biomarkers corresponding to disease or a novel therapeutic treatment. By pooling small molecule data from multiple organisms, it is possible to establish nominal metabolite levels and fluctuation patterns over time for a given set of conditions. The use of metabolomics for temporal analysis of bone healing throughout the period of bone repair and remodeling offers insight into the cellular processes that dominate bone physiology. As such, the assessment of these metabolites is of great interest for research aimed at identifying potential diagnostic, therapeutic, and prognostic variables. This is highly applicable in the context of bone regeneration research, particularly as applied to assessing novel osteobiologics, since baseline metabolite profiles that can be used to evaluate key biomarker fluxes within the micro-environment of the repair site. The further tracking of these profiles over cross-sectional time points within a study may elucidate small molecule profiles that could serve as a predictors of bone healing abnormalities. Ultimately, these metabolites might form the bases for clinical algorithms, based on blood tests or tissue samples, that could be utilized to guide early intervention in patients with delayed healing or non-union of bone injuries (18, 25, 26).

In the current study, we establish a baseline portfolio through combinatorial use of traditional radiographic and histologic data coupled with small molecule distribution profiles for bone repair in a caprine tibia segmental defect model. Volumetric trauma in bones was established and observed over the course of 12 months, with samples subjected to both traditional metrics and metabolite profiling at 3-month intervals. At each time point, biopsies were taken from defect centers, and UHPLC-HRMS based metabolomics was then performed to determine unique metabolic profiles associated with bone repair. Data mining of these profiles was then conducted to establish trend patterns that could be indicative of the repair processes observed in both radiographic and histological data. Candidate small molecules biomarkers were then incorporated into a portfolio to be utilized in future bone defects studies for this species to determine if novel materials composites impair or enhance features in the maker panel.

## 2. Methods

### 2.1. Goat tibia segmental defect model

All experimental procedures and protocols were approved by the appropriate animal care and use committees (University of Tennessee IACUC #2383). Mixed breed female goats, 2–6 years old and with mean body weights 52.49 with standard error (SE) ± 0.25 kgs (35–78 kgs) were purchased from a licensed vendor. Goats were group housed in pens, offering free access to grass, hay, water, and trace minerals, and were fed a daily total mixed ration (27). Animals were acclimatized for a minimum of 10 days before surgery. A segmental defect was created and the reparative progression of bone response at 3-, 6-, 9-, and 12-month post-surgical time points was evaluated. Each time point included 8 goats, with a total of 32 study animals.

Goats were withheld from feed for 24 h, and water for 12–18 h prior to surgery. Each goat was sedated with xylazine (0.05 mg/kg IV) and induced into general anesthesia using midazolam (0.25 mg/kg) and ketamine (5 mg/kg). Goats were then intubated, placed into dorsal recumbency, and general anesthesia was maintained using isoflurane gas vaporized in 100% oxygen. Balanced anesthesia was carried out using biometric parameters (heart rate, respiratory rate, temperature, response to stimulus) and isotonic fluids were provided at 10 ml/kg/hr. The right hind limb suspended, clipped, and aseptically prepared for surgery. A 20 cm linear incision was made over the cranial medial aspect of the tibia. The periosteum was incised and stripped from the surface of the bone to simulate trauma and expose the tibia. An 8-hole/4.5-mm thick, locking plate (Custom design plate, Veterinary Orthopedic Implants, St. Augustine, Fl., USA) was applied to the cranial medial aspect of the tibia, with each of the locking screw holes (four at each plate end) positioned to ensure the section of plate spanning the defect was solid and capable of preventing bending failure. Four 4.0 mm diameter locking bone screws were placed into each of the proximal and distal segments of the tibia. A 2.5 cm length full thickness segment was removed using an osteotomy saw and the resulting gap was left unfilled. The subcutaneous tissues were closed with 2–0 polydioxanone in a continuous pattern and skin was closed using 0 polypropylene in a continuous pattern.

A combination splint bandage was placed on the limb to limit limb flexion and protect the surgery site during recovery and for 60 days after surgery. The splint bandage included a thick, padded bandage that incorporated a rear limb shaped splint (Tarsal Real Leg Quick Splint, Large, Jorgensen Laboratories, Loveland, Co., USA) that extended from a point immediately proximal to the metatarsophalangeal joint to a point immediately distal to the femorotibial joint. Each goat received flunixin meglumine (1 mg/kg, IV; twice daily for 3 days), and ceftiofur sodium (2.2 mg/kg, IV; once daily for 3 days). Additional pain control was provided via a fentanyl transdermal patch (50 mcg/hr.), placed on the dorsal lateral thorax 18 hours prior to surgery and continued for 3 days after surgery. Analgesics were administered (meloxicam 1mg/kg, orally; once daily, and/or fentanyl transdermal patches, 50 mcg/hr) as needed based on pain score. The goats had free access to food and water post-operatively and were monitored for morbidity daily. Splint bandages were changed daily for the first 5 days, followed by every other day for 2 weeks, then weekly for 2 months, at which point bandages were removed. Animals were housed in individual pens for the first 7 days post-operatively, then group housed in pens thereafter. Goats were humanely euthanized at 3-, 6-, 9-, or 12-month intervals by intravenous overdose of a barbiturate (pentobarbital, 1cc/10lbs). All described assessment of defects and collection of samples began immediately following euthanasia and were performed within 1 h postmortem (samples for metabolomic assessment were stored at −80°C to preserve sample integrity).

### 2.2. Radiographic imaging

Post-operative digital radiographs (two images: dorsal to plantar and lateral to medial projection) were performed immediately following surgical procedures, with subsequent imaging carried out monthly until predetermined end points (NEXT Equine DR, Sound, Carlsbad, CA, USA). Radiographs were scored by a board-certified radiologist to assess ostectomy gap filling by radiopaque tissue (Table 1). For statistical analysis, a score of five was equated with a healed fracture (ostectomy gap completely filled with radiopaque tissue and/or bridging callus present on all cortices).

**Table 1:** Radiographic scoring criteria.

**Ostectomy gap filling**	**Score**
No interval change compared to immediate post-operative radiographs	0
New % mineral radiopacity <25% of the ostectomy gap	1
New % mineral radiopacity 26–50% of the ostectomy gap	2
New % mineral radiopacity 51-75% of the ostectomy gap	3
New % mineral radiopacity >75% of the ostectomy gap	4
Ostectomy gap completely filled and/or bridging callus present on all cortices	5

### 2.3. Dual energy X-ray absorptiometry

Dual energy x-ray absorptiometry (DEXA) (Hologic QDR 4500, Horizon DXA Systems, Marlborough, MA, USA) was performed immediately postoperatively, with subsequent scans taken monthly until end points. Briefly, goats were sedated (xylazine 0.05 mg/kg IV) and placed in sternal recumbency. Hind limbs were extended, and the right hind limb was scanned using the lumbar spine settings. A region of interest (ROI) was drawn over the osteotomy gap and bone mineral content (g) and bone mineral density (BMD) (g/cm^2^) were calculated.

### 2.4. Computed tomography

Following euthanasia, locking plates were removed and goats placed in dorsal recumbency to facilitate computed tomography (CT) scans (Philips Brilliance-40, Philips International B.V., Amsterdam, Netherlands). Transverse images were reconstructed with 2 mm slice thickness to generate sagittal and dorsal multiplanar (MPR) images.

### 2.5. Biomechanical evaluation

Following CT scans, tibias were harvested, isolated, and prepared for 4-point bending mechanical testing. Loading was carried out using an Instron 5965 electromechanical universal testing system (Instron 5965, Norwood, MA., USA) fitted with a 5kN actuator and performed as single load-to-failure samples. Load points were optimally positioned such that the ostectomy site was centered at midpoint between both the internal and external arms. The external mount arms served as holding grip for samples and was set to 170 mm, while the internal loading arms were set to 70 mm separation distance. Testing was performed at a rate of 60 mm/min until the specimen failed; maximal load for actuators was set at 61 kgf. The displacement of the actuator was measured as a relative distance (mm) of original location at the start of the test. Tissue specimens lacking sufficient integrity to be mounted and tested were considered to have a null score for the purpose of statistical analysis.

### 2.6. Histochemical and morphometric characterization

Tibia segments were trimmed to remove excess tissue and were fixed in 70% ethanol. Using an automated tissue processor (ASP300S, Leica, Germany), samples were dehydrated in a series of ethanol solutions of increasing concentration (70, 80, 95 x2, 100% x3) at ambient temperature and a programmed auto-cycle of pressure, vacuum, and gentle agitation. Tissues were next transferred to three separate exchanges of 100% Methyl Salicylate (Sigma-Aldrich, St. Louis, MO) for 48–72 h and manually cycled between gentle agitation and vacuum up to −20 mm-Hg (Fisherbrand, Vacuum Chamber). Once fully dehydrated, specimens were transferred to 100% xylenes (Sigma-Aldrich, Histological Grade, St. Louis, MO) for a quick rinse before being placed back onto the automated tissue processor for three changes of 100% xylenes to complete the tissue clearing and prepare for methyl methacrylate resin infiltration. Samples were transferred through three separate and fresh in-house prepared infiltration solution exchanges of methyl methacrylate (Sigma-Aldrich, St. Louis, MO) and dibutyl phthalate (Sigma-Aldrich, St. Louis, MO), under ambient temperature over the course of 1.5–2 weeks, with manual cycling between gentle agitation and vacuum up to −20 mm-Hg. Following resin infiltration, specimens were transferred to polypropylene containers, into which a final resin solution was then added along with a benzoyl peroxide-based catalyst (Perkadox-16, AKZO Nobel Chemicals, Chicago, IL) to initiate polymerization. This resulted in clear, bubble-free hardened methyl methacrylate (MMA) blocks over a period of approximately 5–8 days. These sample blocks were trimmed using a wet bandsaw (MarMed Bone Wet Band Saw) so that resulting microtome sections would fit onto 50 × 75 mm glass microscope slides (Fisherbrand). Sections were cut at 5 μm thickness using a motorized SM2500 sledge microtome (Leica, Germany), fitted with d-profile (sledge) tungsten-carbide knives (Delaware Diamond Knives), and were mounted individually to 50 × 75 mm glass microscope slides (Fisherbrand), coated with an in-house gelatin-based solution recipe (Haupt's Solution), and covered with plastic protective strips.

Slide-mounted sections were then allocated for Von Kossa, Goldner's Trichrome, or Tartrate Resistant Acid Phosphatase (TRAP) staining. Prior to all stains, sections were deplasticized to fully exposed tissue surfaces to staining reagents.

Quantitative assessment of the percent surface area mineralization (Von Kossa), osteoid formation (Goldner's Trichrome), and osteoclast number (TRAP staining) was facilitated by processing digitized image captures from a Panasonic HC-V770 (8M, 3264x2448, aspect ratio 4:3, extra optical zoom 20x) using ImageJ (28). For percent surface area mineralization and osteoid, binary masks of images were generated such that saturated pixels (either mineral content in VonKossa or osteoid in Goldner's Trichrome) could be represented in relation to total image pixels. To determine osteoclastic activity in TRAP-stained slides, osteoclasts per mm of bone surface were counted under brightfield microscopy (29).

### 2.7. Metabolomic analysis

During the previously described tibia trimming for preparing tissue for downstream histological embedding and sectioning, a portion of each tissue sample was dedicated to metabolomic analysis. These samples were immediately processed by collecting 5–6 biopsies from across the defect region to ensure that small molecule profiles are reflective of the entire repair site. For more mineralized samples, a bone chisel was utilized to disrupt tissue for collection. Tissues were weighed for downstream normalization, with weights ranging from 50 to 90 mg, and stored at −80°C. As a final preparation step, samples were pulverized by subjecting tissues to liquid nitrogen and using a mortar and pestle. During this process all samples were maintained on dry ice to avoid thawing. A total of 90 samples were generated across 3-, 6-, 9-, and 12-month time points at *n* = 30, *n* = 20, *n* = 20, and *n* = 20 samples respectively. These replicates therefore represent both internal variance within a given tibial sample and the variance observed within the time point group. After this processing, samples were subjected to metabolite extraction and identification.

Samples were processed at the Biological and Small Molecule Mass Spectrometry Core (BSMMSC), University of Tennessee, Knoxville, TN (RRID: SCR_021368). Water-soluble metabolites were extracted using a previously validated acidic acetonitrile extraction procedure (30). An untargeted metabolomics analysis was preformed using UHPLC-HRMS. Reverse phase ion-pairing chromatography was accomplished by using a Synergi 2.6 μm Hydro RP column (100 mm × 2.1 mm, 100 Å; Phenomenex, Torrance, CA) and an UltiMate 3000 pump (Dionex) with a water:methanol solvent system and tributylamine as an ion pairing reagent. Following elution from the column, metabolites were ionized using negative electrospray ionization, then mass spectral analysis was performed on a Thermo Scientific Exactive Plus Orbitrap (San Jose, CA) operating in full-scan mode (31, 32). Raw files were converted to .mzML files using MSConverter, an open-source software by ProteoWizard (33). Metabolites were identified by exact mass (±5 ppm) and retention time using Metabolomics Analysis and Visualization Engine (MAVEN) and an in-house library of metabolites (34). Area under the curve was integrated for detected metabolites, and intensities were normalized initially based on sample weight. However, difficulties in extracting metabolites from more mineralized tissues presented potential complications with inter-group comparisons. To address this, individual metabolite intensities were normalized by the sum of all detected metabolite intensities for samples in each time point group. This secondary normalization resulted in group distribution profiles that could be directly compared. Normalized data were filtered by interquartile range, log transformed, and Pareto scaled using MetaboAnalyst 5.0 (35). This software was used to generate partial least squares discriminant analysis (PLS-DA) plots. Heatmaps were generated using R (version 1.0.153), which display log2 fold changes and *p*-values calculated by a Student's *t*-test. Online pathway databases were then used to elucidate correlations and select biomarker candidates for a predictive panel.

### 2.8. Statistical analysis of *in vivo* and *ex vivo* data

Statistical analyses were performed using separate mixed-model repeated-measures analysis of variance within the GLIMMIX procedures of SAS to determine whether each outcome variable differed significantly by treatment group over time, with goat considered a random effect (SAS v 9.4, Cary, NC). Multiple comparison adjustments were made using Tukey's *post-hoc* comparisons to assess the effects of group, time point, and group x time point interaction. For categorical variables, ordinal multinomial logistical regression doing a cumulative logit was performed by monthly analysis to determine if there were differences between treatments. Significance was set at *p* < 0.05 and p-values for significant relations are reported. Least square means are reported ± SE.

## 3. Results

### 3.1. Radiographic imaging

**Figure 1:**
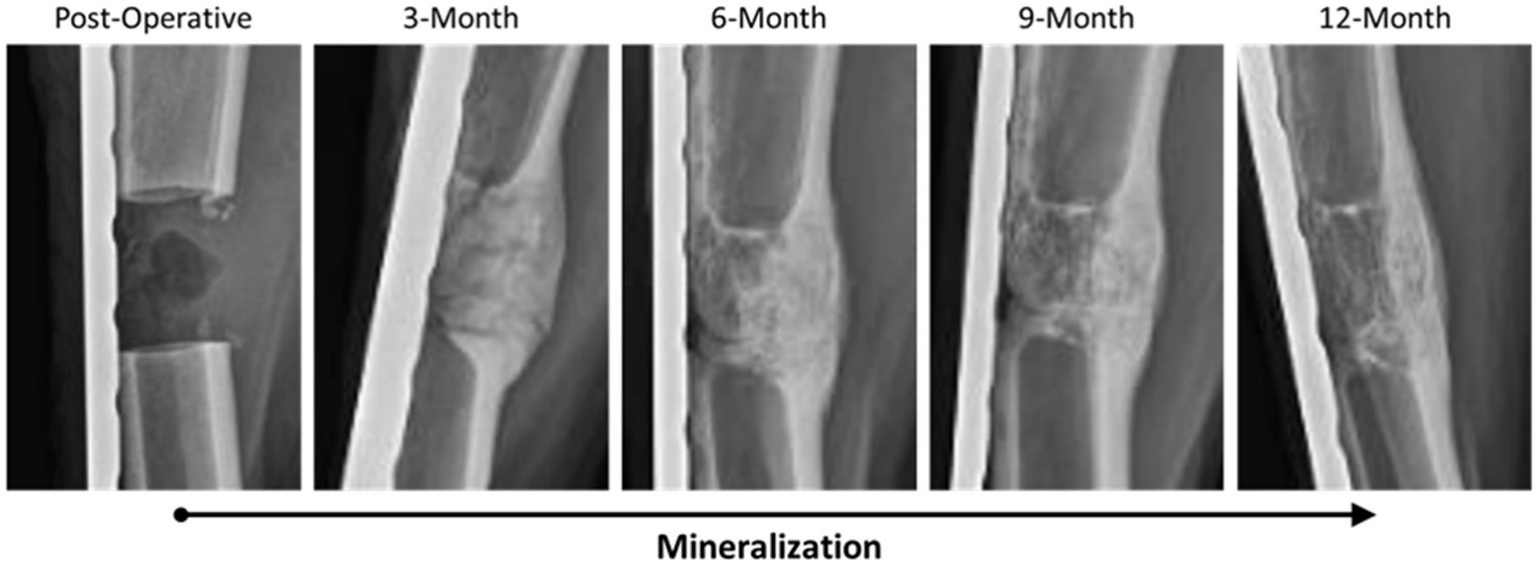
Representative radiographic imaging of defects over the study period demonstrating an increase in mineralization and overall remodeling as a factor of time.

Radiographs for each goat were reviewed and scored based on ostectomy gap filling over time points, with a complete bridging callus forming in 73% of defects. Time was determined to be a significant factor for increasing scores (*p* < 0.0001). This can be observed in side-by-side panels of radiographs over the study time course (Figure 1).

### 3.2. Dual energy X-ray absorptiometry

**Figure 2:**
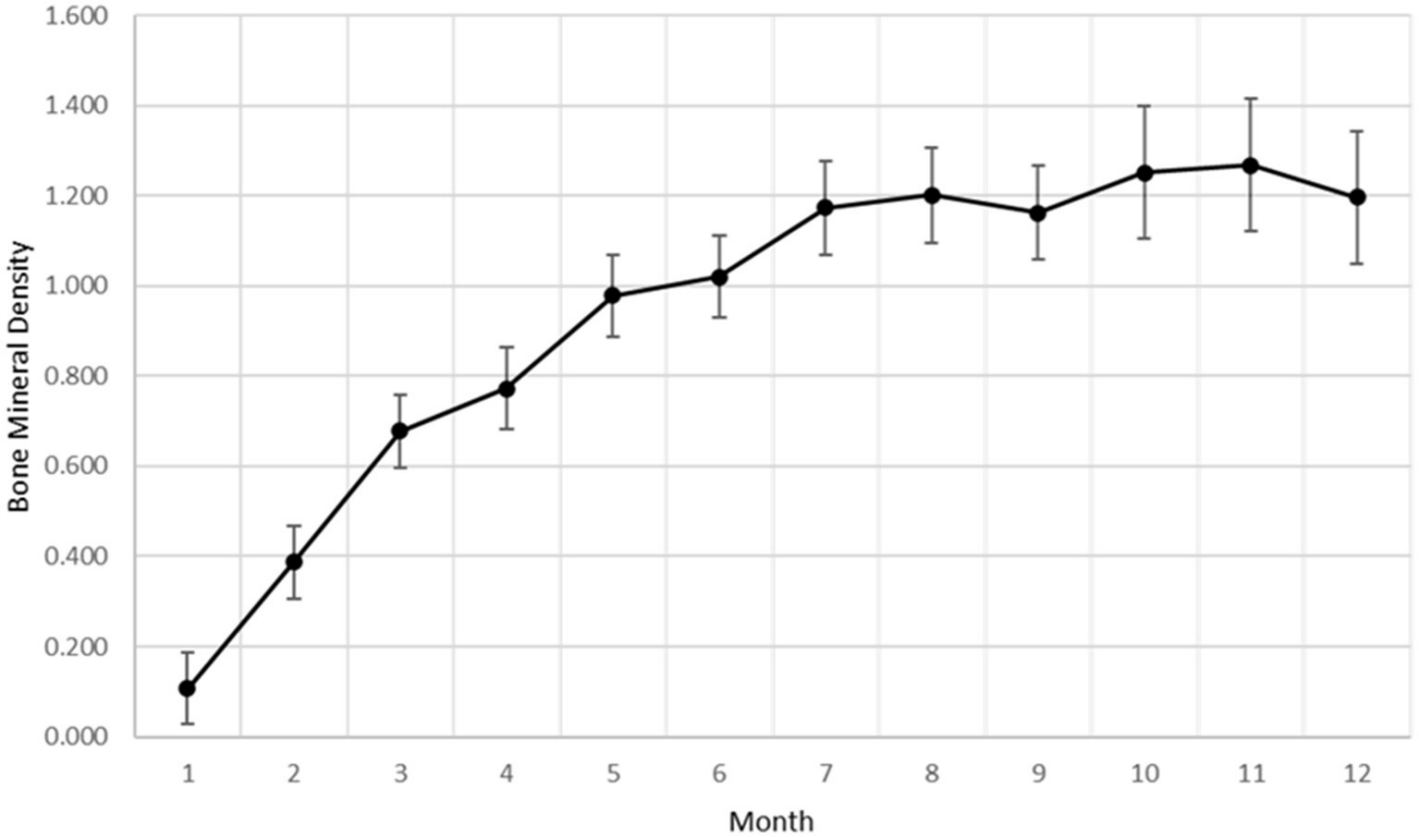
Bone mineral density data as a factor of time generated from DEXA analysis.

Bone mineral density within the ostectomy gap was calculated for each goat at monthly intervals. As with subjective radiographic scores, a significant effect of time (*p* < 0.0001) was established for increasing BMD within the ostectomy gap (Figure 2). The overall rate of change for BMD showed similarities to a quadratic rate for bone healing.

### 3.3. Computed tomography

**Figure 3:**
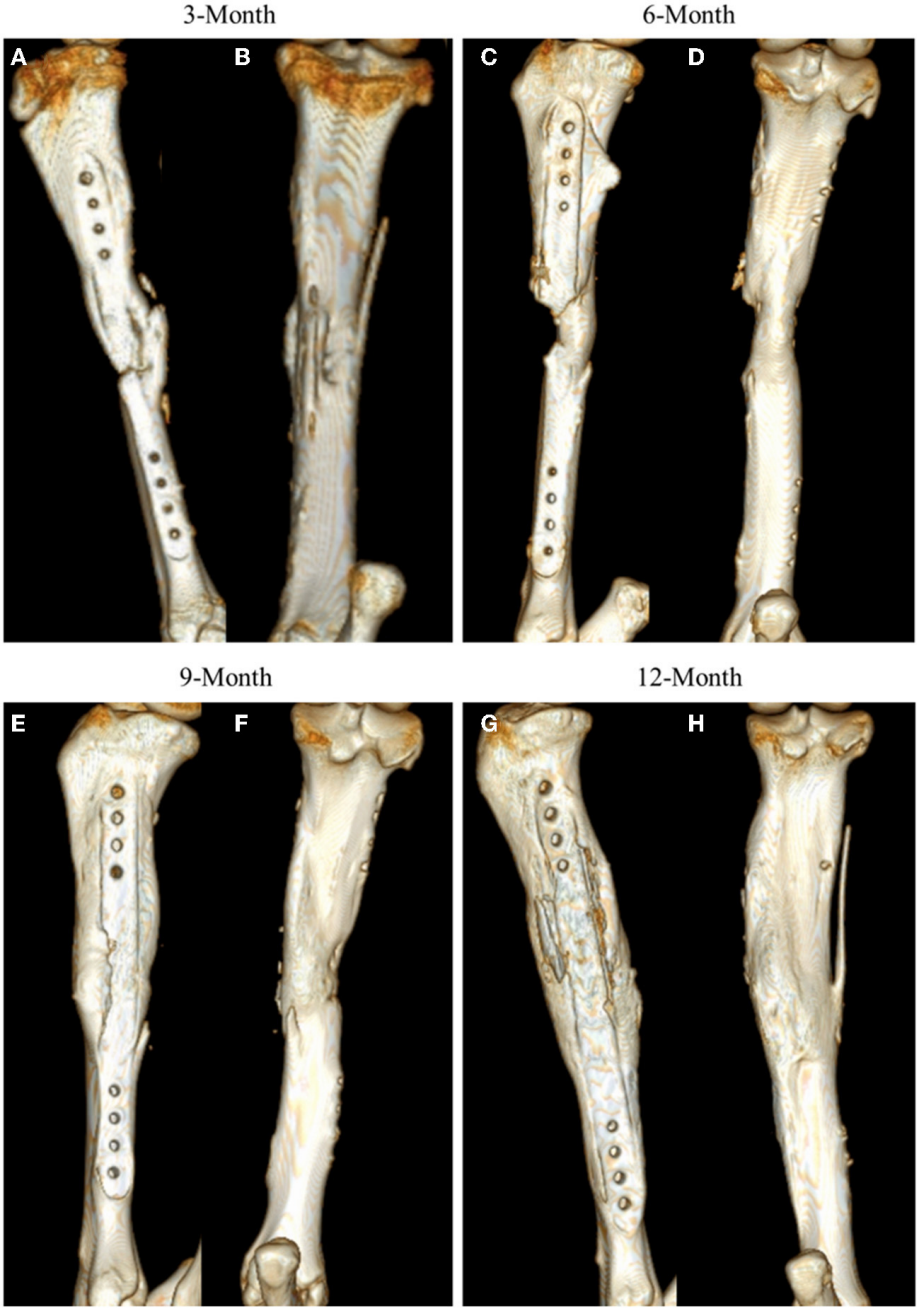
Representative CT 3D renderings for samples at 3-, 6-, 9-, and 12-month time points. CT renderings are displayed in medial facing **(A, C, E, G)** and posterior facing **(B, D, F, H)** angles to highlight mineral content at the site.

As with other radiographic analyses, a significant effect of time was observed (*p* = 0.0037), with indication of new bone increasing from 3-month samples to that observed in 12-month samples (Figure 3).

### 3.4. Biomechanical testing

**Figure 4:**
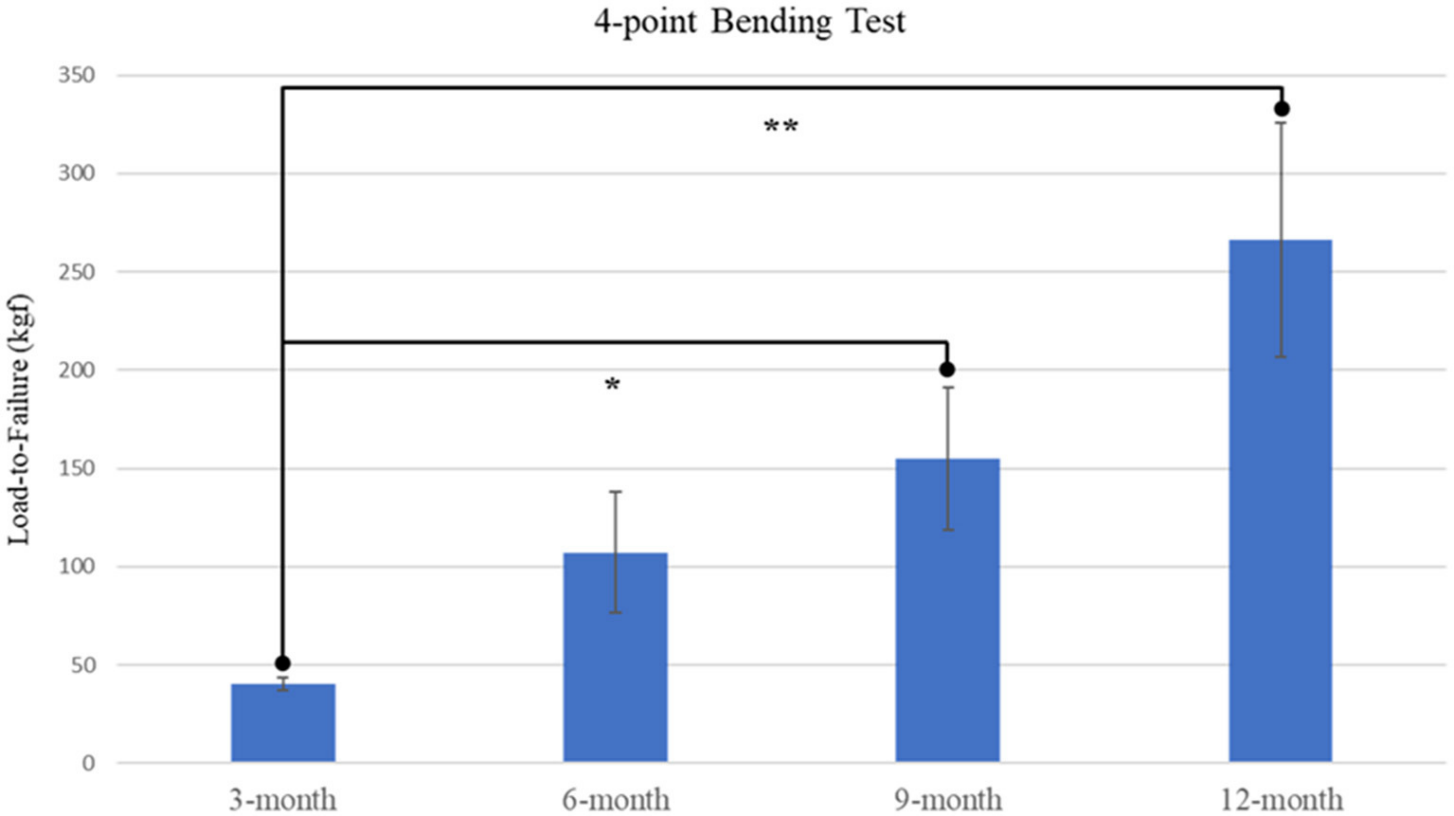
Plot of load-to-failure forces for samples at 3-, 6-, 9-, and 12-month time points. Significant differences observed between 3-month and both 9-month and 12-month samples are denoted by * and ** respectively.

4-point bending load-to-failure testing was performed in nineteen of twenty-six (73%) goats, with remaining tissue samples lacking sufficient integrity to be mounted and examined. A significant effect of time was determined (*p* = 0.0126) with increased loading capacities displayed in both 9-month and 12-month samples as compared with 3-month samples (Figure 4). It was observed that no statistical difference existed between mechanical loading capacity of samples after complete callus bridging of the ostectomy gap, with mean specimen failure values of 142.10 kgf (SE ± 25.19 kgf).

### 3.5. Histochemical and morphometric characterization

**Figure 5:**
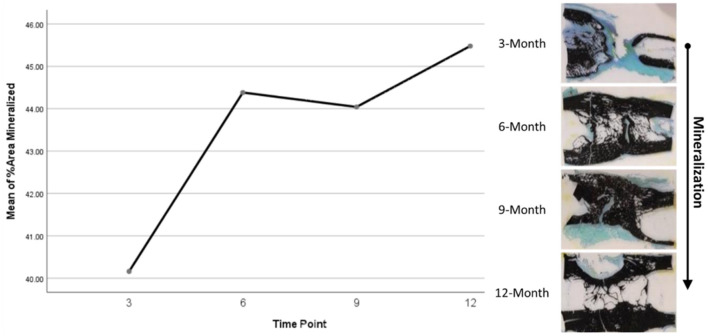
Representative Von Kossa histological staining and plotted mineralization data.

**Figure 6:**
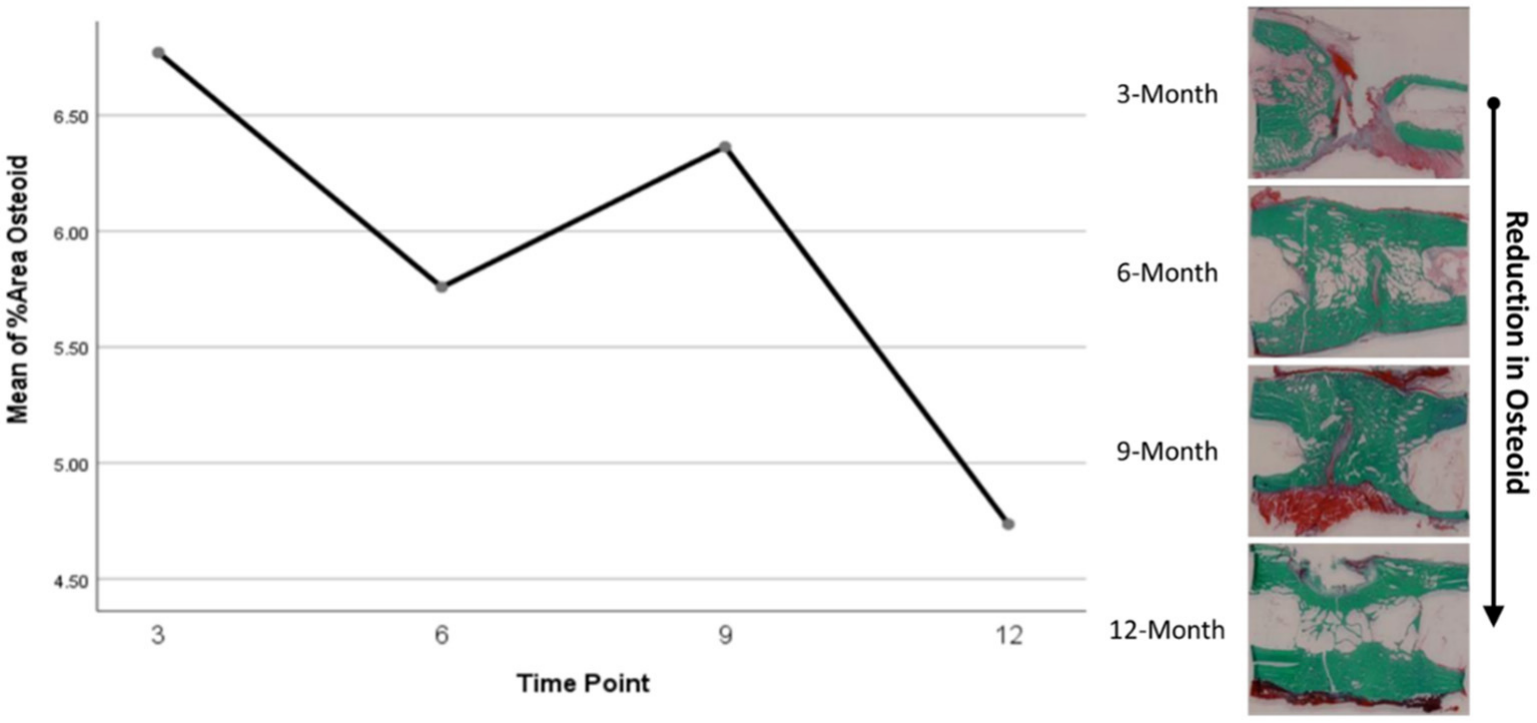
Representative Goldner's Trichrome histological staining and plotted osteoid content data.

Histomorphometry assessment of Goldner's Trichrome staining found that the osteoid percentage of area in the ostectomy gap decreased over time, with a slight increase at 9 months then final decrease at 12 months. This significant difference over time (*p* = 0.0359) supports an active ostectomy remodeling gap regardless of group that mimics the percent mineralization in an equal and opposite direction in that new bone (osteoid) is initially laid down, mineralized, and then remodeled and decreases over time. Histomorphometry assessment of Von Kossa staining, as an indicator of percent area mineralized in the ostectomy gap, found that the percent mineralization was similar across time points (41%, SE ± 1.92%). While no significant difference was apparent over time, the mean percent mineralization over time appeared to follow a bone remodeling curve in that mineralization initially increased (new bone was laid down), decreased as the ostectomy gap remodeled and then proceeded to increase again (Figures 5, 6). Analysis of TRAP-stained samples revealed that mean osteoclast counts per mm of bone surface remained were similar across time points (for all time points, 0.10 osteoclasts/mm bone area, SE ± 0.80).

### 3.6. Metabolomic analysis

**Figure 7:**
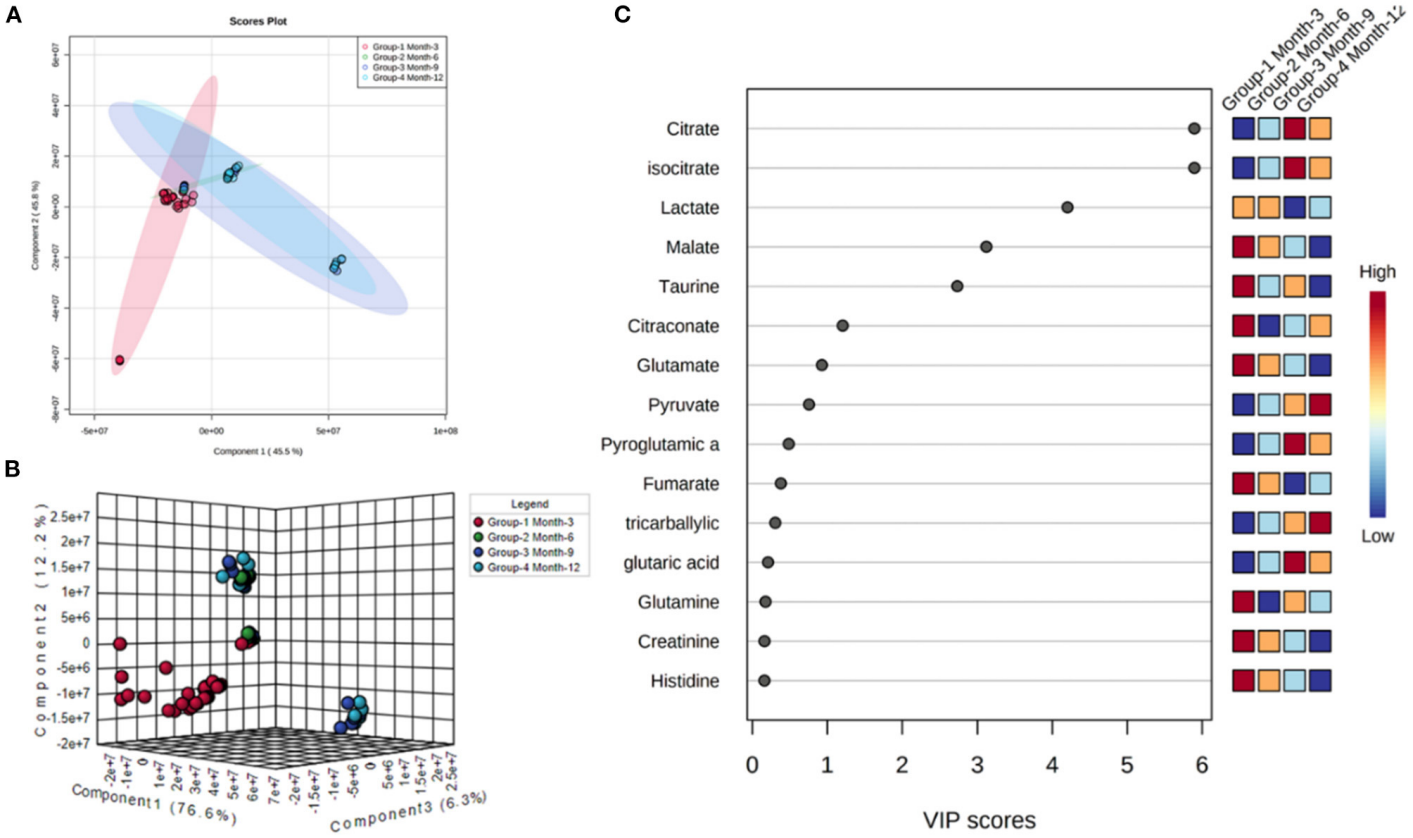
2D **(A)** and 3D PLS-DA **(B)** plots showing separation of small molecule profiles for samples with groups color coded to designate 3, 6, 9, and 12-month groups. VIP score chart **(C)** illustrating metabolites driving group separation across study period.

**Figure 8:**
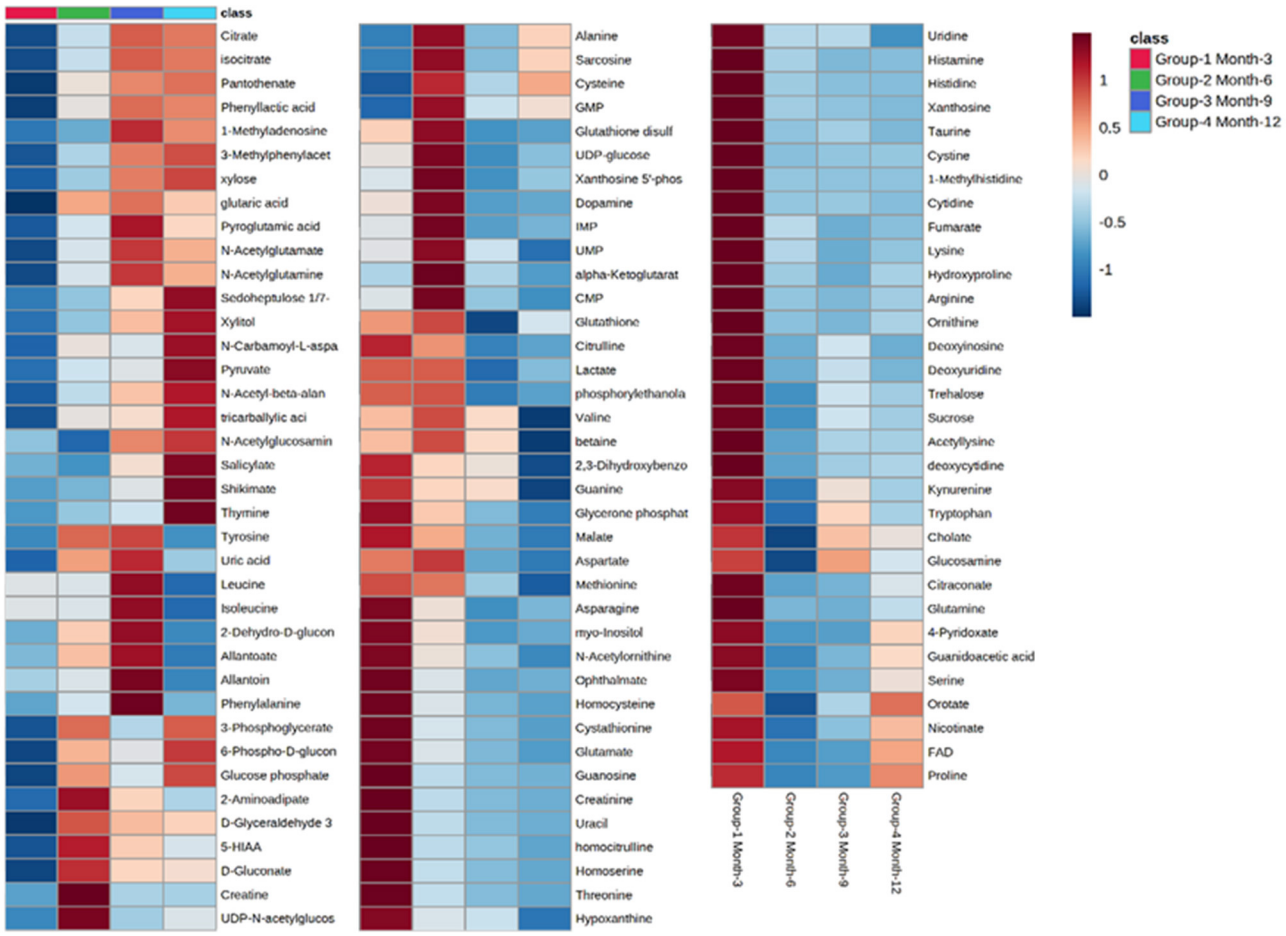
Heatmap illustrating abundance profile fluctuations in detected metabolites over study time points. From left to right, columns denote 3, 6, 9, and 12-month time points, with each row representing a detected metabolite.

Initial assessment of metabolite relative abundances for samples was performed using Metaboanalyst, an online platform designed for user-friendly metabolomic analysis. 2D and 3D partial least squares-discriminant analysis (PLS-DA) plots, as well as corresponding metabolite variable importance in projection (VIP) scores, were generated to examine sample clustering and separation based on sample timepoint (Figure 7). 3-month sample clustering was observed to be separate from subsequent time points, indicating that the metabolic profiles of these early-stage samples were unique and distinct. Metabolite VIP scores demonstrated that citrate/isocitrate, lactate, malate, and taurine contribute most to the observed separation of groups in PLS-DA plots, with a VIP score > 1 indicating that a given metabolite is significant in driving group separation. This was supported by the heatmap generated for normalized metabolite intensities within groups that demonstrated distinct molecular pattern shifts over the study time course (Figure 8).

**Figure 9:**
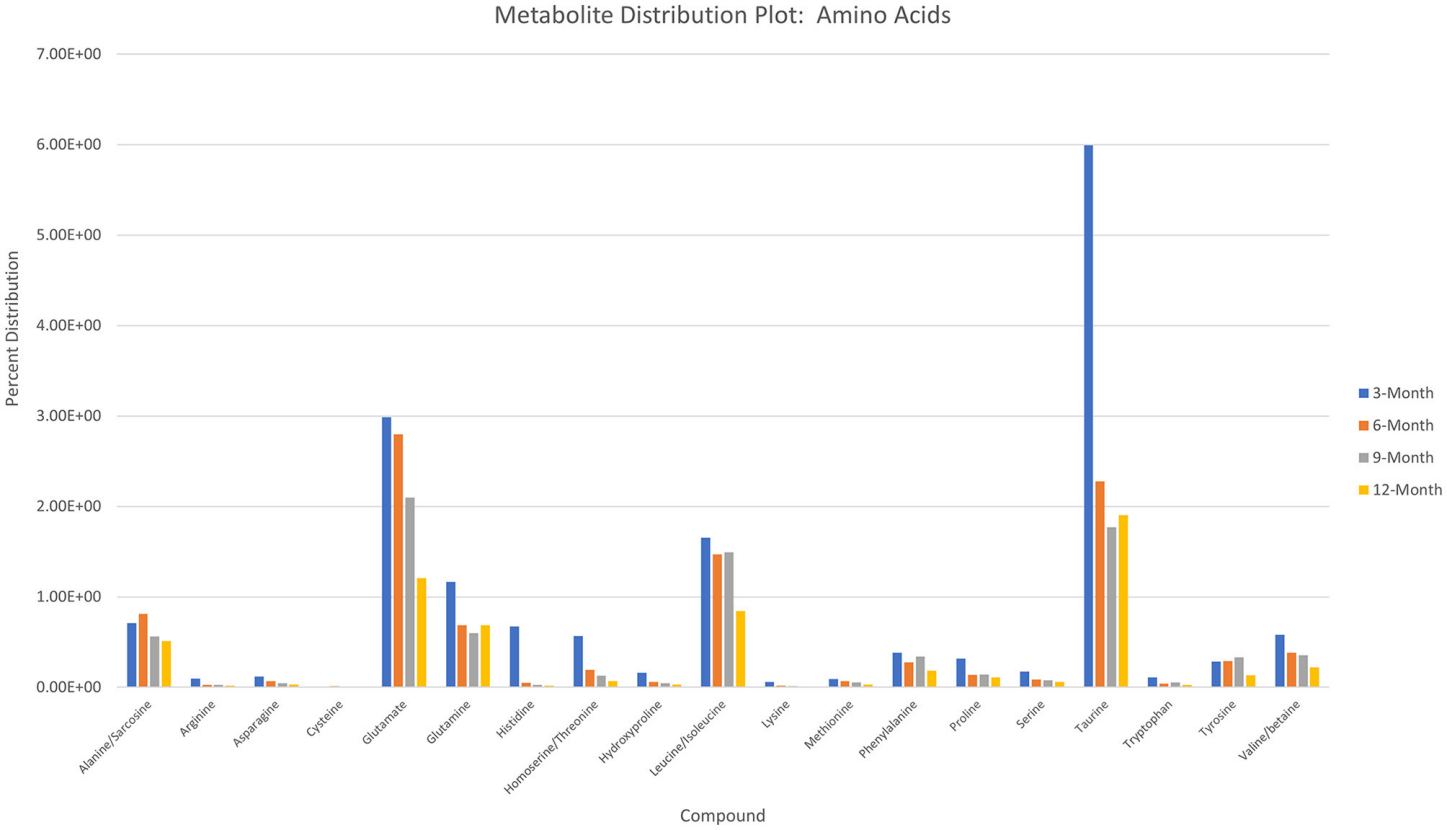
Histogram depicting normalized metabolite abundances for essential, non-essential, and conditionally essential amino acids detected in all samples.

**Figure 10:**
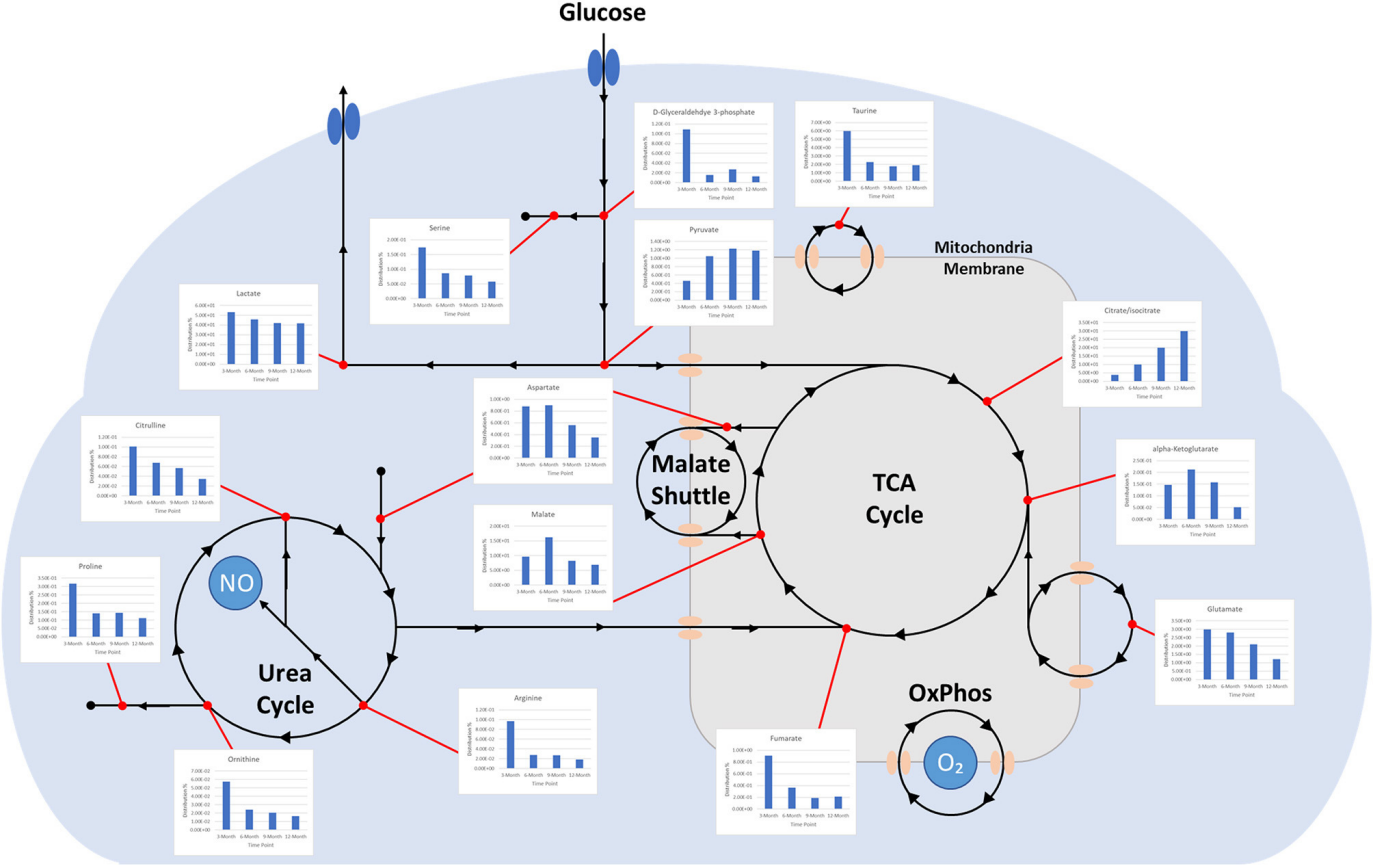
Diagram depicting time-dependent small molecule normalized abundances as related to glycolysis. (1) Early enhanced lactate levels are indicative of conditions conducive to osteoblastic differentiation and activity. (2) Early enhanced levels of arginine may be indicative of angiogenic activity through the production of nitric oxide. (3) Early enhanced proline levels are indicative of collagen deposition to form soft callus tissue. (4) Increasing citrate/isocitrate and pyruvate levels are indicative of a switch to the TCA cycle for energy production and subsequent mineralization of tissue. (5) Decreasing taurine levels are indicative of more conducive environment for osteoclastic differentiation and may represent a move toward more homeostatic conditions.

Charts generated based on the normalized metabolite abundance data for sample groups showed profile fluctuations for metabolites over time points. From these data, essential, non-essential, and conditionally-essential amino acid profiles were isolated to illustrate the reduction in building block molecules over time (Figure 9). To contextualize normalized metabolite abundance profiles that appeared to be directly related to bone repair and remodeling processes, a diagram depicting the glycolytic pathway and related energy metabolic functions was fabricated and overlain with metabolite distribution charts (Figure 10).

## 4. Discussion

The use of a long-term volumetric defect models that resemble closely the normal fracture healing process following trauma is of great interest for the field of bone tissue engineering. Such regeneration model studies are lacking, especially for application and evaluation of novel synthetic biomimetic materials. The extensive characterization required for meeting FDA and ISO 10,993 standards further demonstrate the need for cost-effective large animal preclinical models. Establishment of a robust and streamlined model may both expedite the development period for and effectiveness of novel medical devices for bone tissue engineering, providing superior graft technologies for reducing rates of non-unions, need for amputations, or prevalence of costly secondary revision surgeries.

Based on radiographic and histologic data from this model, the volumetric bony defects observed callus formation to bridge the segmental gap, followed by mineralization. This repair and remodeling process is demonstrated by the increasing density readings and opacity on DEXA and radiographic imaging respectively, with confirmation from histologic evaluation of Von Kossa and Goldner's Trichrome stains. Von Kossa stain, which binds silver ions to calcium in boney tissue, highlighted mineralized bone content in samples. Goldner's Trichrome staining, a modified general bone and cartilage stain similar to Masson's Trichrome staining, contrasted bone and soft tissue morphology, with dense collagen/osteoid presenting as red and mineralized bone as green. As the ostectomy gap observed in 3-month histological samples already demonstrated tissue in-growth and osteoid content, it follows that the bulk of the soft callus formation and initialization of mineralization must have occurred during this initial period. The compiled data from 3-month to 12-month time points therefore represents the mineralization phase, characterized by heighten osteoblastic activity, and subsequent remodeling phase, in which osteoblastic activity is dampened and recruitment of osteoclasts is initiated to counterbalance mineral deposition. This provides an essential contextual landscape for the performed small molecule analysis.

Incorporating UHPLC-HRMS based metabolomics as a supplementary assessment mechanism offers the potential to establish a biomarker panel that correlates to the observed bone remodeling in this large volumetric defect. This would constitute a valuable reference pattern to assess data taken from similar models in which treatments such as graft technologies or other therapeutics are utilized, and would afford a more direct means of objective comparisons between dissimilar therapeutics. Sample collection was performed immediately following bulk extraction of the defect site and preserved at −80°C within 1 h postmortem to prevent degradation of analytes (36). To better compare the small molecule intensity data between different time points, in which increasing mineral content made metabolite extraction more difficult, the values associated with any given small molecule were normalized to present as a percentage of the overall metabolite distribution profile in addition to an initial normalization by sample weight. As indicated by metabolite VIP scores, the small molecules lactate, citrate, malate, taurine, and glutamate were observed to be the major factors driving group separation for time points, all of which play key roles as intermediates and products in glycolytic energy production. For this reason, glycolysis, the TCA cycle, and amino acid profiles were the primary focus of this assessment.

Well-documented for its presence and role in early stage wound response, lactate levels in samples demonstrated a steady decrease across sequential time points. Lactate has been described as a potential driver in bone repair and it abundantly produced by pro-inflammatory immune cells that are initially recruited to the injury. Increased levels within the initially formed hematoma have been observed to both recruit mesenchymal stem cells and induce angiogenic functions in endothelial cells, as well as stimulate collagen deposition for establishing new extracellular matrix architecture (1). Furthermore, lactate is considered to be important for supporting osteoblastic differentiation from precursor cells, as glycolysis is the primary energy mechanism utilized by mature osteoblasts (37). Gene pathways, such as Wnt pathways, act on the glycolysis engine *via* mTORC2 to enhance osteoblast differentiation by regulating the critical junction molecule pyruvate and promote the conversion to lactate, as compared with entry into the mitochondria for the TCA cycle (38, 39). Despite the inefficiency of aerobic glycolysis, it has been speculated that the dependence on this metabolic energy source may be related to the resulting abundance of intermediate metabolites that can aid in biosynthesis activities or act as antioxidants, preventing an environment that may favor adipogenesis (40). This would account for the relatively high initial levels of lactate observed in samples with decreasing levels denoting a reduced demand for enhanced osteoblastic activity, potentially indicting a change in the tissue environment toward a more homeostatic condition (1).

Supporting this are the profiles for pyruvate and citrate, which both demonstrate dramatic increases in relative abundance over the study period. This would indicate a metabolic shift to the TCA cycle and reduced conversion of pyruvate to lactate (18, 27). Citrate has been described as a critical molecule for bone biochemical stability and decreased content has been observed with disease-linked bone loss (41). As a major product molecule of the TCA cycle, citrate is closely associated with high energy production and plays a key role in the formation of apatite nanocrystals during osteoblast activity, making the molecule critical for mineralization (42, 43). For these reasons, this metabolite has observed particular attention as a potential additive for biomaterial technologies aimed at enhancing bone repair and has been proposed as a possible dietary supplement (44, 45). The time dependent increase in citrate levels observed in this study therefore may serve as a direct representation of increasing bone mineral content within the defect, which would complement radiographic data. Beyond simply mirroring mineral levels, 3- and 6-month values may also indicate an increasingly conducive environment for osteoblastic activity, as these samples were largely soft tissue when harvested.

Apart from citrate, several intermediates of the TCA cycle were also detected, including malate, α-ketoglutarate, fumarate, and succinate, with both malate and α-ketoglutarate demonstrating increased distribution levels at 6 months before decreasing over the remaining study period. Combined with the values for detected essential, non-essential, and conditionally essential amino acids, which all observed decreased abundances over the study period, this may represent an initial energy demand during the early-stage repair to establish extracellular matrix architecture and stimulate osteoblastic activity that is then followed by reduced energy demands when approaching more homeostatic conditions. In particular, the presence of proline at the early stages of repair would be essential as it is a precursor molecule for collagen synthesis and therefore required for extracellular matrix formation (46). Amino acid levels would therefore present as consumables in this energy production and as demand decreases, distribution profiles for these molecules would similarly decrease (47–50). 6-month increases for malate and α-ketoglutarate may be the result of external metabolic elements, such as the utilization of glutamate or malate-aspartate shuttle activity. The latter of these mechanisms is of interest due to its connection with arginine metabolism, which is essential for production of nitric oxide during conversion to citrulline. Nitric oxide has been described as a critical regulator of bone anabolism through modulation of glycolysis in osteoblast precursors to promote osteoblastic differentiation, as well as stimulator of angiogenesis to provide nutrients and oxygen to newly forming tissues (51).

Potentially operating in a supporting role, taurine was found to have initially high levels that then decreased over the study period. While the precise actions of taurine during bone repair are still unclear, it has been demonstrated to promote ALP activity and mineralization in MG-63 populations and demonstrated beneficial effects on bone *in vivo* (52). There is evidence that taurine concentrations may play a unique regulatory role in stabilizing the pH sensitive boundary of the mitochondrial membrane and allows for enhanced biosynthesis and energy production capabilities (53). This would account for part of its role in osteoblastic influence since these cells maintain high energy demand during function. Notably, this metabolite has also been described as a protective agent against oxidative stress-induced apoptosis and as a means for inhibiting the Wnt antagonists SOST mRNA and Sclerostin protein, which would further enhance osteoblast differentiation while impairing osteoclast differentiation (54). The observed decrease over the study period of both taurine and its precursor methionine may therefore again indicate a shift away osteoblastic activity and toward a more homeostatic remodeling environment.

Osteoclastic activity, like that of osteoblasts, require large amounts of energy in order to produce required enzymes for effectively degrading established bone matrix. However, osteoclasts primarily depend on oxidative phosphorylation during differentiation, as compared with glycolysis used by osteoblasts (55). This differentiation of monocyte precursors is facilitated by the factors M-CSF and RANL, and results in a multi-nucleated cell capable of lysing bone extracellular matrix. The small molecule pantothenate, which was observed to increase and plateau over the course of the study, has displayed a potential dual effect on RANKL-induced osteoclastogenesis, with lower concentrations stimulating increased differentiation and higher concentrations inhibiting differentiation (56). The observed metabolite distribution profile therefore may represent an activation phase for osteoclasts to serve remodeling roles, followed by an inhibitory phase to stabilize the remodeling region and move toward a homeostatic environment. This may be supported by the sharp increase of shikimate levels at the 12-month time point. Shikimate acts as an anti-inflammatory molecule, inhibiting nociception by TNF-α and PGE2, and many of its derivatives are associated with promoting osteoblastogenesis, with some also inhibiting TNF-α-induced osteoclastogenesis (57, 58). In contrast with differentiation, the main energy mechanism for mature and active osteoclasts appears to be glycolysis rather that fatty acids or ketones. The increase in pyruvate over time may similarly be an indicator of sustained glycolytic activity throughout the stages of repair.

These small molecule data when coupled with radiographic and histologic records appear to illustrate a soft callus environment in the 3-month samples that undergoes increased mineralization to form a hard callus environment subject to both osteoblastic and osteoclastic remodeling agents by 9- and 12-month time points. More specifically, they highlight the energy sources utilized for cell recruitment, differentiation, and primary activity during primary tissue reconstruction and downstream remodeling. Though speculative, metabolic profiles heavily reinforce the traditional data to generate a more comprehensive view of bone repair in this model. As described by Fan et al., such metabolic signatures serve as a potent tool for truly characterizing bone regeneration post-traumatic injury (18, 59).

## 5. Conclusion

In this study we established a large segmental bone defect in goats that reflects trauma resulting in substantial volumetric bone loss. Characterization of the native repair capacity was observed for up to 12 months with sampling at 3-, 6-, 9-, and 12-month time points. Data at each time point consisted of traditional radiographic (X-ray imaging, DEXA, and CT) and histological information, as well as UHPLC-HRMS metabolomic profiling. Increases in radiographic opacity and scoring indicated that later time points contained more new bone, which was also reflected in callus formations under histologic evaluation. Small molecule profiles generated for all samples found distinct patterns associated with bone tissue repair. In particular early-stage spikes in lactate levels and elevated amino acid levels indicated an environment conducive to osteoblast differentiation and extracellular matrix formation, while increasing citrate and pyruvate levels indicate enhanced accumulation of mineral content within the defect region. Furthermore, initial spikes in taurine and late-stage spikes in shikimate and pantothenate appear to represent a shift toward a more homeostatic environment through the recruitment and differentiation of osteoclasts for callus remodeling. As sampling from the contralateral limb for controls was not performed for metabolomic analysis, the current study is limited in its capacity to display a comprehensive metabolomic base profile. These control samples would be of great interest for continued research. A fundamental understanding of these molecular patterns is critical to addressing the existing knowledge gap surrounding physiological bone repair following major trauma over time. A comprehensive metabolic portfolio can then be applied as a baseline reference when examining the effectiveness of novel biomaterials and serve as guide for the development of new targeted therapeutics.

## Data availability statement

The original contributions presented in the study are included in the article/supplementary material, further inquiries can be directed to the corresponding authors.

## Ethics statement

The animal study was reviewed and approved by the Institutional Animal Care and Use Committee.

## Author contributions

AB, RR, and CP were responsible for data generation, analysis, writing, formatting, and critical scientific review of data. CC, RG, and SH were responsible for data generation, analysis, and critical scientific review of data. SA, P-YM, HC, SC, and DA were responsible for providing critical scientific review of data and as key scientific advisory for direction on experiments. All authors contributed to the article and approved the submitted version.
